# Utility of Fetal Cardiac Resonance Imaging in Prenatal Clinical Practice: Current State of the Art

**DOI:** 10.3390/diagnostics13233523

**Published:** 2023-11-24

**Authors:** Alice Pozza, Elena Reffo, Biagio Castaldi, Irene Cattapan, Martina Avesani, Roberta Biffanti, Annachiara Cavaliere, Alessia Cerutti, Giovanni Di Salvo

**Affiliations:** 1 Pediatric Cardiology Unit, Department of Women’s and Children’s Health, University of Padua, 35122 Padua, Italy; 2 Department of Cardiovascular Imaging and Dynamics, Katholieke Universiteit Leuven, 3000 Leuven, Belgium; 3 Pediatric Radiology, Neuroradiology Unit, University Hospital of Padua, 35128 Padova, Italy

**Keywords:** fetal cardiology, fetal cardiac magnetic resonance imaging, congenital heart disease

## Abstract

The field of prenatal cardiac imaging has revolutionized the way we understand and manage congenital heart diseases (CHD) in the developing fetus. In the prenatal period, cardiac imaging plays a pivotal role in the diagnostic pathway, from screening to classification and follow-up of CHD. The ability to visualize the fetal heart in utero allows healthcare professionals to detect abnormalities early, thus enabling timely interventions and informed decision-making processes for both the mother and the medical team. Early CHD detection improves preparation for delivery, postnatal care, and postnatal outcomes. Advancements in medical technology and imaging techniques have provided clinicians with insights into the fascinating workings of the fetal heart. Several imaging modalities have proven to be helpful in this field, with echocardiography undoubtedly representing the primary modality for evaluating the fetus. By providing detailed anatomical and functional information, fetal cardiac magnetic resonance (CMR) imaging contributes to better prenatal counseling and enhances the coordination of care between obstetricians, maternal–fetal medicine specialists, and pediatric cardiologists. Shortcomings of fetal CMR are due to technical concerns related to the intrauterine position of the fetus and subsequent challenges to following a standard scan protocol. The aim of this paper was to revise the current state-of-the-art in the field of fetal CMR and its clinical applications and to delve into methods, challenges, and future directions of fetal CMR in prenatal imaging.

## 1. Introduction

In the last 20 years, fetal cardiology has developed into a highly specialized clinical field that joins together perinatal medicine and pediatric cardiology [1,2,3]. This discipline has acquired growing interest thanks to advancements in medical imaging technologies, thus enabling healthcare practitioners to delve deeper into the structure and function of the fetal heart. Early detection of CHD is crucial, as cardiac defects are the most common birth anomalies, affecting approximately 1 in every 100 infants worldwide. Early diagnosis allows for appropriate interventions and management strategies before birth, during delivery, and in the postnatal period [3,4]. Fetal cardiology provides parents and healthcare providers with vital information about the fetal heart’s health, enabling them to make informed decisions about pregnancy, delivery, and subsequent care of the newborn [5].

Echocardiography has traditionally played a crucial role in fetal cardiology for assessing anatomy, cardiac function, blood flow patterns, and heart rhythm. Improvements in ultrasound technologies have made it possible to define in detail most forms of CHD, even in the late first trimester [1,2].

To optimize the diagnostic value of fetal ultrasound cardiac imaging, P. Quaresima et al. [3] emphasized adherence to a standardized scan protocol, according to guidelines from major scientific societies.

Recently, strain echocardiography (speckle tracking or tissue Doppler-based strain and strain rate) was proposed to increase the diagnostic abilities of fetal imaging. By tracking the movement of distinct points within the heart muscle over the cardiac cycle, myocardial deformation parameters help in understanding fetal heart mechanics. Di Salvo et al. [4] demonstrated that strain by speckle tracking provides information about the contraction and relaxation of different segments of the fetal heart in order to identify subtle changes in myocardial function in cardiomyopathies and CHD that might not be apparent using traditional echocardiography alone. To study fetal arrhythmias, strain from tissue Doppler should be preferred because it offers a higher frame rate (up to 300 fps, three times higher than speckle tracking) [5,6]. Finally, three-dimensional (3D) echocardiography addresses the capture of complete spatial information by acquiring multiple two-dimensional images from different angles and reconstructing a 3D representation of the fetal heart, which allows an enhanced visualization of complex cardiac anomalies [7,8].

Despite significant progress in fetal echocardiography, technological advances and ongoing experience have enhanced our ability to evaluate fetal heart disease, with growing interest in the use of cardiac magnetic resonance (CMR) imaging [9]. Like echocardiography, CMR is safe for both mothers and fetuses. Fetal CMR offers the potential to obtain important additional information about fetal cardiovascular physiology and to provide an alternative modality for defining the cardiac anatomy and fetal hemodynamics, which might be useful when ultrasound (US) imaging is hampered by a poor acoustic window [10].

## 2. Hardware Consideration

To perform fetal CMR, the first key decision is to choose between 1.5 Tesla (T) and 3 T machines. Both 1.5 T and 3 T magnetic resonance (MR) machines emit strong magnetic fields and radiofrequency pulses to create detailed images. Thus, contraindications are the same as for any MR examination: mothers implanted with pacemakers, cardioverter defibrillators, or any MR-incompatible device must be excluded before scanning [11,12,13]. Concerns might arise regarding potential risks to the developing fetus due to the strong magnetic fields and possible heating effects [14,15], although potential increased risks based on higher magnetic fields were not demonstrated by [16]. In addition, MR machines are nowadays designed with various safety features, such as gradient-induced acoustic noise reduction and maternal and fetal monitoring, with the straightforward purpose of enhancing safety during pregnancy.

The higher magnetic field generated by a 3 T machine might increase signal-to-noise ratios and spatial resolution, achieving higher image quality [9]. This might be helpful for visualizing small fetal cardiac structures and for adding details about cardiac morphology [17]. In addition, fetal CMR at 3 T enables comprehensive functional assessment, including evaluation of blood-flow patterns, ventricular volumes, and myocardial tissue characterization [18]. Higher magnetic field strengths can lead to shorter scan times while maintaining image quality [19].

However, a 1.5 T CMR offers a safe and accessible tool when assessing CHD and fetal cardiac function. While it may have some limitations in spatial resolution compared to higher field strengths, it is often preferred for imaging pregnant patients due to its weaker magnetic field [20]. The weaker field strength reduces the likelihood of potential heating effects on the fetus and decreases the risks associated with strong magnetic field exposure [15]. Geiger et al. [21] reported that fetuses examined at 1.5 T obtained a significantly higher imaging quality than at 3 T because of lower sensitivity to motion artifacts and fewer inhomogeneities of the radiofrequency field. In conclusion, 1.5 T CMR is usually sufficient to perform fetal CMR. Higher magnetic fields might be used to address specific clinical queries or for research purposes.

To perform fetal CMR, a dedicated coil is not necessary, and a simple, commonly available phased-array torso surface coil is usually employed. Mothers should preferably lie in the supine decubitus position; left lateral decubitus is also possible. Fetal visualization is first achieved with localizer sequences, and to optimize signal intensity of the fetal region, the fetus should be positioned within the coil center. If the signal intensity is not adequate, the coil must be repositioned accordingly. Scout sequences are obtained in the three orthogonal planes of the mother’s abdomen (axial, sagittal, and coronal) using 6–8 mm single-shot fast spin-echo (SSFSE) T2-weighted sections with a 1–2 mm gap and a large field of view (320–400 mm) [21].

Fetal CMR protocol consists of static and cine imaging to investigate both anatomical and functional characteristics of the fetal heart. Static imaging of the fetal heart includes balanced steady-state free precession (bSSFP) and SSFSE (also defined as *black blood*) sequences such as half-Fourier acquisition single-shot turbo-spin echo (HASTE). Due to their high spatial resolution and short acquisition time, these sequences allow whole fetal body evaluation for extracardiac anomalies, precise depiction of macroscopic anatomy, and detection of mediastinal vascular abnormalities. The drawback of static sequences is that they provide gross anatomic information but no functional data. CINE-bSSFP sequences with cardiac gating are employed to acquire more precise anatomic information, while also adding functional assessment thanks to their high spatial and temporal resolution. The dynamic evaluation of the fetal heart with these kinds of sequences is multiplanar, scanning the fetal thorax on the axial, coronal, and sagittal planes and the fetal heart in the four-chamber and short-axis views. The scan time of cine imaging is longer than that of static imaging, thus possibly being affected by respiratory artifacts and fetal movements. Multiple studies reported that scan time with such a study protocol varies between 30 and 60 min [22,23].

Fetal CMR aims primarily to quantitatively evaluate blood flow in the principal vessels. In this regard, some studies have demonstrated the viability of 2D phase-contrast imaging in fetuses of late gestational age. These sequences, however, are highly affected by fetal motion artifacts and require long scan times. To overcome this evergreen challenge when performing MR scans, 4D-flow imaging has been employed to acquire a comprehensive blood-flow examination (for example, to evaluate shunt lesions or to investigate venous and valvular flow) [24,25]. In everyday clinical practice, 4D-flow has been revealed as a promising tool that needs some improvements to overcome the relatively low spatial and temporal resolution and the extreme sensitivity to motion and respiratory artifacts. Actually, 4D-flow remains the most interesting way to deeply investigate blood flow phenomena of fetal circulation [26].

The high complexity and large amount of information provided by fetal CMR should be processed by an expert and experienced cardiologic and radiologic team.

## 3. Application of Fetal CMR Imaging in Understanding CHD

Magnetic resonance imaging is gaining greater importance in the field of fetal cardiology.

First of all, it should be considered in cases of poor echocardiographic acoustic window or in the presence of concomitant extra-cardiac defects with an indication for MR, like esophageal atresia, fetal diaphragmatic hernia, lung malformations, and airway blockages [20,22].

In addition, image quality of fetal CMR is not affected by oligohydramnios, late gestational age, maternal obesity, multiple gestations, placental position, or progressive calcification of the ribcage in late-gestation patients [11,21,27]. Fetal CMR can be a valuable diagnostic tool in cases of intrauterine growth restriction (IUGR), a condition in which a fetus fails to achieve its expected growth potential in the womb. In cases of severe IUGR, the fetus might be at risk of cardiac dysfunction due to inadequate oxygen and nutrient supply. In this regard, fetal CMR provides a well-established technique for measuring blood flow, which may add additional hemodynamic information beyond Doppler assessment of blood velocity [17]. Fetal CMR allows information about qualitative imaging biomarkers to be obtained, with the final purpose of understanding the biological processes of cardiovascular fetal programming [28].

Furthermore, it is useful in the assessment of the fetal cardiovascular system, from measurement of ventricular volumes and ejection fractions to the quantification of cardiac output, systemic and pulmonary blood flow, valvular regurgitation, volumes and percentages, and intra- and extracardiac shunt volumes and directions, as well as the parametric analysis of myocardial microstructure and the determination of oxygen content in blood vessels [26,29,30,31,32]. Monitoring cardiac function helps healthcare providers make decisions about time and set of delivery and future management.

CMR allows non-invasive assessment of ventricular diastolic and systolic volumes for both ventricular chambers. It clearly identifies endocardial and epicardial contours, both for the left and right ventricles. In addition, cine CMR sequences can be used to measure right and left ventricular myocardial mass. In a fetal animal model, Schrauben et al. used CMR to study heart functioning. By using bSSFP, the authors derived ventricular volumes from short-axis views. Unfortunately, the methods used in the afore-mentioned study cannot be used for fetal CMR in humans. Moreover, despite assessment of ventricular volumes and functional systolic parameters described, studies were limited to a few case reports of small case series [33]. In conclusion, functional assessment is not considered a common indication to perform a fetal CMR in clinical practice.

**Figure 1:**
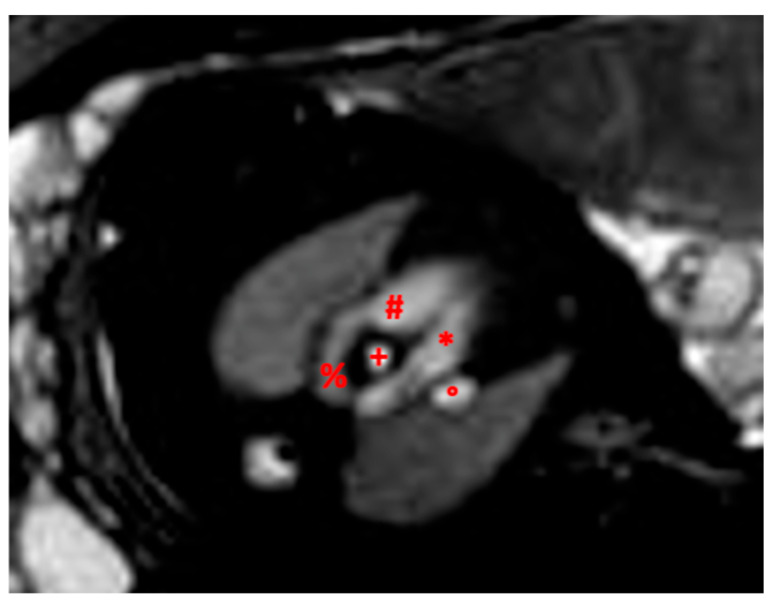
Example of fetal CMR image. Image of a 33 gestational week fetus demonstrating right aortic arch (*); superior vena cava (°); and trachea (+); (#) left pulmonary artery; (%) left patent ductus arteriosus.

On the other hand, fetal CMR can offer details on great vessel anomalies like aortic arch anomalies, vascular rings, aberrant left subclavian artery, retro-aortic innominate vein, or bilateral superior vena cava [10,33] (Figure 1). Most importantly, several papers demonstrated that CMR is superior to echocardiography for diagnosing aortic coarctation and aortic arch anatomy [33,34]. It provides detailed, three-dimensional images of the fetal heart and blood vessels, assessing the extent and location of the aortic coarctation. It offers better spatial resolution and better visualization of structures surrounding the aorta, which can aid in diagnosing associated anomalies or complications. Whereas fetal echocardiography relies heavily on the operator’s expertise and experience to obtain high-quality images, CMR is less operator-dependent and may yield consistent results regardless of the operator’s experience. Future studies should evaluate the opportunity to use CMR to select patients to be referred to a third-level center rather than allow delivery in a peripheral hospital.

CMR can be useful in conotruncal diseases, in particular to better define pulmonary artery anatomy. CMR can be considered in fetuses affected by tetralogy of Fallot with unusual anatomies, like pulmonary valve atresia or suspected pulmonary branch(es) discontinuity, or in cases of major aorto-pulmonary collateral arteries (MAPCAs). To diagnose these cardiac defects, fetal echocardiography remains the cornerstone of prenatal assessment. Nevertheless, it is not accurate to visualize the afore-mentioned unusual anatomies, which is, however, possible with fetal CMR. For example, CMR detects if one pulmonary branch originates from the patent ductus arteriosus (e.g., pulmonary artery discontinuity). Such a finding plays a crucial role in postnatal management, as the newborn can benefit from immediate treatment with endovenous prostaglandin infusion to ensure adequate pulmonary perfusion until pulmonary ductus arteriosus stenting.

To better identify the type of truncus arteriosus (see van Praagh or Collett–Edwards classifications); in particular, to rule out pulmonary artery discontinuity and to better understand the anatomy of the aortic arch, CMR is of great value [35,36].

**Figure 2:**
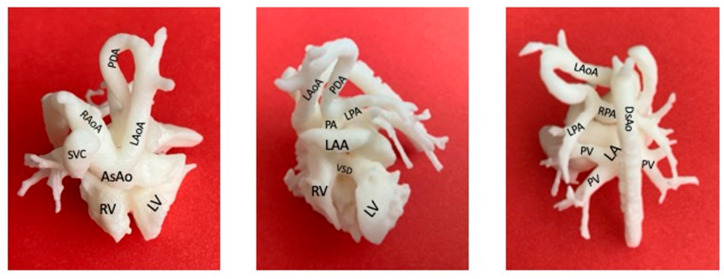
3D printing of a 28-gestational-week fetus with double-outlet right ventricle pulmonary atresia. Double aortic arch, interrupted left aortic arch after PDA origin. PDA-dependent pulmonary blood flow, patency of distal pulmonary trunk, pulmonary arteries. Left atrial appendage juxtaposition. (On the left: cranial view. In the middle: anterior view. On the right: posterior view.) RV: right ventricle; LV: left ventricle; SVC: superior vena cava; LA: left atrium; LAA: left atrial appendage; RAoA: right aortic arch; LAoA: left aortic arch; PDA: patent ductus arteriosus; VSD: ventricular septal defect; PA: pulmonary artery; LPA: left pulmonary artery; RPA: right pulmonary artery; PV: pulmonary vein, DsAo: descending aorta; AsAo: ascending aorta) (Courtesy of Materialise, Leuven, Belgium).

Joshua et al. [32] depicted fetal CMR as a promising tool for 3D representation of the fetal heart in a workflow of slice-volume registration and post-processing volume reconstruction using a dedicated software. Motion-compensated cine CMR sequences have the potential to measure ventricular volumes and track longitudinal ventricular growth in this setting [37,38]. Reconstructions confirm that correct spatial and temporal features are reliably visualized, suggesting that CMR acquisition and framework reconstruction has the potential for volumetric fetal heart assessment [39,40]. Finally, 3D reconstructions might be used by software for post-processing, like virtual anatomy, augmented reality, and 3D printing (Figure 2) [41]. These anatomical details are very important for planning surgery, identifying variants at higher risk of associated genetic/chromosomic disorders, and obtaining more effective parental counseling [42]. Overall, 3D heart models enhance parental counseling by improving communication, understanding, and decision-making regarding CHD. They provide a hands-on tool for medical professionals to increase parents’ awareness about their child’s heart defect, as they offer a powerful visual aid that can ease the emotional burden on parents while ensuring that they are well-informed about their child’s condition and treatment options [43].

An additional application of fetal CMR is in the field of cardiac masses; using weighted-specific sequences, it allows tissue typing of an intracardiac mass, depicting whether it mainly consists of muscle tissue, fibers, or fat [44,45]. Vachon-Marceau et al. [46] reported that CMR is helpful in fetuses referred for massive cardiac rhabdomyoma, as the lesion can benefit from transplacental treatment with sirolimus, leading to a significant reduction in the volume of the mass and an improvement in biventricular systolic function, thus affecting future surgical treatment [47,48]. In addition, tissue definition of intracardiac lesions by using CMR might address further investigations after birth, like genetic tests, in the case of rhabdomyomas, which are, conversely, excluded in the presence of teratomas, fibromas, etc.

Fetal CMR techniques have been developed for measuring blood flow and intravascular and intracardiac blood oxygen saturation, which implement hemodynamic information otherwise not available through fetal echocardiography [26,49,50]. Flow quantification and blood oxygen determination allow the implementation of knowledge on fetal cardiac physiology and provide insight into the impact of cardiac pathology on fetal circulation, brain perfusion, and oxygen distribution [31,51].

Current technological developments in the field of cardiac imaging have allowed a comprehensive understanding of CHD, thus optimizing surgical management [52,53]. Fetal CMR has shown a promising role in the planning of fetal cardiac interventions: in utero balloon dilation of semilunar valves early in the second trimester has been performed with the aim of increasing the chances of achieving a postnatal biventricular circulation [54,55]. Ongoing experience has documented that in utero atrial septum perforation, balloon dilation, or stenting are now feasible. Nevertheless, larger multi-centric or randomized studies should be planned to confirm the safety/effectiveness profile of in-utero procedures for complex CHD [56]. Marini et al. [57] explored the potential of fetal CMR to provide additional diagnostic information and treatment options in the setting of invasive in utero cardiac and vascular interventions. Other authors have confirmed the ability of CMR to identify pulmonary lymphangiectasia secondary to a restrictive atrial septum in hypoplastic left heart syndrome. Those patients may greatly benefit from fetal atrial septostomy [58,59].

## 4. Challenges of Fetal CMR

In pediatric and adult populations, a combination of cardiac gating and breath-holding or respiratory navigation is used to guarantee diagnostically useful image quality [9]. The transfer of these concepts to fetal cardiovascular assessment is difficult because of the small size of the fetal cardiovascular structure within a large field of view, short duration of the fetal cardiac cycle, fetal gross stochastic movements, and high rate of maternal breathing [60]. During examination, maternal breath-holds are brief and ideally avoided to optimize maternal comfort. First, to achieve high-quality cardiac imaging, it is mandatory to synchronize the cardiac cycle with the CMR. After birth, it can be easily obtained by using an ECG gating, while it is unavailable in fetuses [61,62,63]. Geiger et al. [21] demonstrated that non-gated dynamic steady-state free-procession CMR sequences allow for elementary assessment of cardiovascular anatomy. To overcome this limit and to increase to expand the diagnostic capabilities of CMR, alternative methods have been developed.

Metric optimized gating (MOG) retrospectively reconstructs oversampled data by using a post-processing method to identify and correct heart rate abnormalities after scanning [64]. MOG extracts the fetal heart rate by iteratively reconstructing CINE images in post-processing [65]. Self-gated magnetic resonance imaging provides wireless cardiac cycle synchronization involving the use of real-time image acquisition strategies [61,66,67]. This way, clear images can be reconstructed without motion artifacts. Self-gating and MOG are limited by time-consuming post-processing, the need for special (and expensive) software, the inability to adjust for heart rate variations, and relatively long reconstruction times that prevent evaluation of images during MR examination.

**Figure 3:**
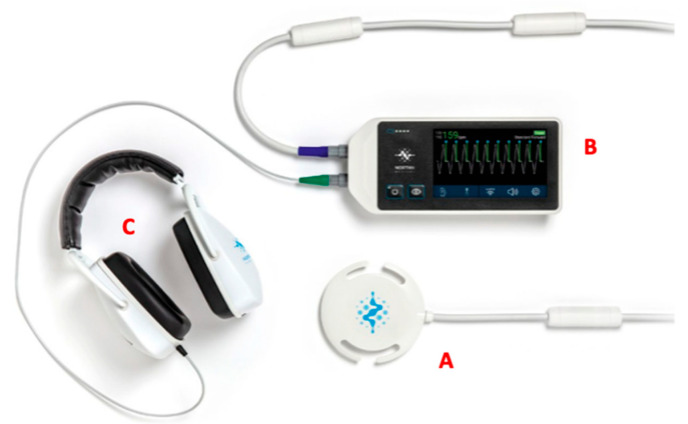
Doppler ultrasound set for fetal CMR imaging. (**A**) Doppler ultrasound interface with cable; (**B**) display; (**C**) headphones for practitioners to detect fetal heartbeat. (Adapted from Fetal smart-sync Flyer, Northh Medical).

The most common technology for measuring fetal heart rate is cardiotocography (CTG), which operates by identifying the dominant frequency in a signal acquired with a non-imaging ultrasound transducer directed at the fetal heart. Typically, CTG returns a time-averaged measure of the fetal heart rate at regular intervals without detecting every single heartbeat of the fetus, which is insufficient for MR gating [68]. Unfortunately, the CTG toll is normally not designed for the MR environment, regarding safety concerns, because it is not MR-compatible. Thus, a MR-compatible device was built ad hoc for this purpose: a Doppler ultrasound (DUS) probe [68,69] (Figure 3). MR-compatible DUS refers to the integration of Doppler ultrasound technology with MR systems, allowing simultaneous or sequential acquisition of both ultrasound and MR data. This combined approach can provide complementary information about anatomical structures and physiological processes, enhancing the diagnostic capabilities of medical imaging. DUS probes have been developed as an alternative to ECG sensors for gating the fetal heart rate and producing dynamic cine images. The probe is placed on an elastic belt set around the maternal abdomen after finding the position with the most consistent fetal cardiac signal by using headphones. This device is directly connected to the MR scanner for data synchronization and produces a dynamic signal, monitoring fetal cardiac cycle motions based on a Doppler waveform [70]. Triggers derived from the waveform are transferred to the MR scanner to synchronize data acquisition. This device is cheap and effective. In addition, a practical benefit of this approach is the ability to use established cardiovascular protocols for fetal imaging with immediate in-line image reconstructions to support clinical work flow. However, several re-positionings due to fetal movements may be required [71].

## 5. Limitations of Fetal CMR

MR offers the potential to obtain important additional information about cardiovascular physiology in fetal patients with CHD and to provide an alternative modality for defining cardiac anatomy, emerging as a challenging tool that is complementary to fetal US in the setting of a suboptimal echocardiographic acoustic window. Nevertheless, this new emerging technique has several limitations.

Fetal CMR imaging is available only in a few tertiary care centers. Special skills are needed to perform and interpret the exam. In addition, the indication should be addressed by a pediatric cardiologist skilled in fetal cardiology, and it requires highly specialized radiologists to perform scan protocols and post-processing image analysis.

Long exam sessions, the need for ECG gating, and repeated sequences make it expensive and time-consuming. Thus, fetal CMR cannot be considered a primary screening tool [29,72]. Image quality of CMR increases with gestational age because the fetus is bigger and the movements are progressively less prominent. Therefore, it should mainly be considered starting from the 25th week of gestation [73]. A higher magnetic field might (partially) overcome these limits. Despite the fact that up to 7 T devices exist, they are not widely available and are mainly used for research purposes. In addition, the safety of such high magnetic fields for fetuses should be stated.

Severe maternal obesity might limit the use of CMR as the patient would be too large for the CMR scanner, and since wider patient tables reduce image quality. In addition, claustrophobia and anxiety can lead to an increase in mothers’ movements during scanning, thus resulting in motion artifacts, which lower a scan’s quality and worsen its diagnostic value [21]. Sometimes scans may need to be stopped, in particular, by pregnant women in most advanced stages of pregnancy, who may not tolerate prolonged immobilization in closed spaces.

Failure of the maternal cardiovascular system to adapt to pregnancy vascular overload might lead to placental dysfunction, resulting in fetal growth restriction (FGR) [74]. Fetal CMR in the context of FGR is challenging, as the small size of fetal structures results in lower spatial resolution, and reduced amniotic fluid might limit image quality. In addition, FGR is associated with increased vulnerability to motion artifacts due to compromised fetal well-being.

Recent studies have sought to determine how maternal factors may modify the risk of fetal CHD by investigating the relationship between fetal heart development and maternal angiogenic imbalance [75]. Pregnancies complicated by preeclampsia indirectly affect the fetus by reducing the oxygen and nutrient supply, potentially leading to FGR and thus impacting heart development [76]. This issue makes fetal CMR applicability challenging.

Despite the fact that there is no clear evidence of fetal injuries or spontaneous miscarriages due to magnetic fields generated by CMR, the exam is not recommended in the first trimester of pregnancy [19]. The Food and Drug Administration (FDA) discourages the use of fetal MR in the first trimester and fixes the specific absorption rate to 4 W/kg for maternal whole-body MR studies, regardless of the magnetic field strength [72,73]. Gadolinium administration in pregnancy is contraindicated due to potential teratogenicity in the first trimester and fetal kidney excretion. Recommendations are to avoid gadolinium-enhanced MR unless essential and consider non-enhanced MR in the first trimester [77].

## 6. Future Perspectives

The heart develops from a tubular framework, which, by a process of looping and torsion, evolves into a four-chambered structure. Despite the fact that the macroscopic heart structure is completed within the first two months of gestation, the heart ultrastructure continues to evolve up to birth and beyond. Mekkaoui et al. [78] studied the myocardial tissue of five hearts from 10 weeks of gestation to birth by using a 3 T CMR. They found that myocardial fibers gradually develop from a single radially arranged layer to a double longitudinal layer at birth. By using layer-specific speckle tracking echocardiography, Castaldi et al. [79] demonstrated that the endocardial longitudinal layer develops first and is well represented starting at 17 gestational weeks, while epicardial longitudinal layer maturation is slower and is completed just at the end of gestation. Functional data were confirmed by histological data. Dysfunctional myocardial fiber development can be found in some CHDs, like hypoplastic left heart syndrome. In these patients, fetal interventions (atrial septostomy or percutaneous aortic valve balloon valvotomy) might invert the progression to univentricular heart physiology. Thus, high-magnetic-field CMR (3–7 T) might be considered a promising tool for studying left ventricular myocardial ultrastructure to increase accuracy of patients’ selection for surgery and to better establish the right timing for the procedure in order to improve the chances of postnatal surgery [53,54].

## 7. Conclusions

As medical science and technology continue to advance, the modern pediatric cardiologist should not just suspect a CHD but rather build a plan for postnatal care. Fetal echocardiography remains the cornerstone of first-line detection of CHD. It is a safe, easy-to-perform, and highly sensitive examination that can be employed starting from the first trimester of pregnancy. Once a complex CHD is diagnosed, further diagnostic tools are available to define, in detail, the anatomy of the heart and great vessels. Fetal CMR can overcome the limitations of US to improve image quality in cases with a poor acoustic window. In addition, blood-flow software and tissue characterization algorithms can offer important details on diagnosis and prognosis of complex and/or rare anatomy.

Nevertheless, the best fetal CMR results are actually achieved almost in the third trimester of pregnancy (26–32 gestational weeks). The future perspective is to move up the timing of fetal CMR scans to early in the second trimester (hopefully, from 18 gestational weeks), keeping the same spatial/temporal resolution and compliance with safety concerns.

Finally, images obtained from CMR can be used for teaching or counseling purposes; they can be 3D-printed or downloaded in virtual anatomy or augmented reality software, opening up new frontiers in diagnostic and interventional cardiology. Technological advancements are expected to enhance image quality, diagnostic accuracy, and clinical outcomes. As research and technology evolve, fetal CMR is likely to revolutionize prenatal care and improve the outcomes of infants born with CHD.

## Data Availability

Not applicable.
